# The Stability Improvement of *Aspergillus fumigatus α*-Amylase by Immobilization onto Chitin-Bentonite Hybrid

**DOI:** 10.1155/2022/5692438

**Published:** 2022-03-14

**Authors:** Ezra Rheinsky Tiarsa, Yandri Yandri, Tati Suhartati, Heri Satria, Bambang Irawan, Sutopo Hadi

**Affiliations:** ^1^Department of Chemistry, Faculty of Mathematics and Natural Sciences, University of Lampung, Jl. Prof. Dr. Sumantri Brojonegoro No. 1, Bandar Lampung 35145, Indonesia; ^2^Department of Biology, Faculty of Mathematics and Natural Sciences, University of Lampung, Jl. Prof. Dr. Sumantri Brojonegoro No. 1, Bandar Lampung 35145, Indonesia

## Abstract

Enzyme immobilization is a powerful method to improve the stability, reuse, and enzymatic properties of enzymes. The immobilization of the *α*-amylase enzyme from *Aspergillus fumigatus* on a chitin-bentonite (CB) hybrid has been studied to improve its stability. Therefore, this study aims to obtain the higher stability of *α*-amylase enzyme to reduce industrial costs. The procedures were performed as follows: production, isolation, partial purification, immobilization, and characterization of the free and immobilized enzymes. The CB hybrid was synthesized by bentonite, chitin, and glutaraldehyde as a cross-linker. The free enzyme was immobilized onto CB hybrid using 0.1 M phosphate buffer pH 7.5. The free and immobilized enzymes were characterized by optimum temperature, Michaelis constant (*K*_M_), maximum velocity (*V*_max_), thermal inactivation rate constant (*k*_*i*_), half-life (*t*_1/2_), and transformation of free energy because of denaturation (Δ*G*_*i*_). The free enzyme has optimum temperature of 55°C, *K*_*M*_ = 3.04 mg mL^−1^ substrate, *V*_max_=10.90 *μ*molemL^−1^min^−1^, *k*_*i*_ = 0.0171 min^−1^, *t*_1/2_ = 40.53 min, and Δ*G*_*i*_ = 104.47 kJ mole^−1^. Meanwhile, the immobilized enzyme has optimum temperature of 60°C, *K*_*M*_ = 11.57 mg mL^−1^ substrate, *V*_max_=3.37 *μ*molemL^−1^min^−1^, *k*_*i*_ = 0.0045 min^−1^, *t*_1/2_ = 154.00 min, and Δ*G*_*i*_ = 108.17 kJ mole^−1^. After sixth cycle of reuse, the residual activity of the immobilized enzyme was 38%. The improvement in the stability of *α*-amylase immobilized on the CB hybrid based on the increase in half-life was four times of the free enzyme.

## 1. Introduction

Enzyme stability is a significant factor for commercial enzymes when applied to the batch process using a stirred-tank reactor at extreme temperature and pH*pH* [1]. Most of industrial enzymes have some problems about their thermal instability when applied in batch processes, including easily soluble in water, denatured at high temperatures, may contaminate with other substances in a reaction, and can only be used once in a process. The thermal instability of the enzyme is due to the loss of equilibrium in the noncovalent bonds [2]. Enzyme stability can be investigated by the determination of half-life and thermodynamic parameters, namely, the thermal inactivation rate constant (*k*_i_) and the transformation of free energy because denaturation (Δ*G*_*i*_) [3,4].

One of the simple methods to improve the enzyme stability, reusability, and properties is by the immobilization. After immobilization, the enzyme becomes more stable, can be easily separated from the substrate, and can be used repeatedly to the new substrate. Nowadays, immobilized enzymes have widely used on an industrial scale because of the following advantages: can be used repeatedly, the product is not contaminated by the enzyme, the reaction is easily controlled, and can improve enzyme stability [5]. Therefore, the use of the immobilized enzymes can reduce the consumption cost. Enzyme immobilization is the process of binding or retaining enzyme molecules onto insoluble material as a supporting matrix [6] divided into organic, inorganic, and hybrid or composite material. Several methods are well known and used for enzyme immobilization such as carrier binding, encapsulation, and covalent binding [7].

The *α*-amylase enzyme is used as a biocatalyst to break the starch molecule down by acting on the *α*-1,4-glucosidic bonds to form maltose, dextrin, or D-glucose [5]. Microbial *α*-amylases are commonly applied in various industries such as detergent, syrup, bread and cake, dairy products, starch processing, animal feed, textile and leather, pulp and paper, candy, sugar, bioethanol, pharmaceuticals, and waste treatment [8]. This enzyme has several advantages, including an increase in production yield, decrease in production costs, controlling starch depolymerization, working at physiological conditions, preventing the formation of side products, lower energy activation, and does not need purification phase [9].

In the present study, the *Aspergillus fumigatus α*-amylase was immobilized onto a chitin-bentonite (CB) hybrid matrix. *A. fumigatus* was chosen as the host enzyme because this fungus does not show any special nutritional needs [10]. Meanwhile, chitin (poly-(*β* (1,4)-2-acetamide-2-deoxy-D-glucopyranose)) is a linear polysaccharide formed by N-acetyl-D-glucosamine monomer in *β*-linkage position [11]. Bentonite is a smectite clay mineral with two and one tetrahedral and octahedral layers [12, 13]. Chitin and bentonite were insoluble in water, has large particle surface area, chemically inert and thermally stable, easily activated, has a well-defined layered structure, has the ion-exchange ability, abundant raw material, economic, and environment friendly [11, 14]. Hybrid matrix has some benefits compared to the processes catalyzed using classic material especially for getting the better properties of the matrix and increasing thermal stability of the enzyme [7]. In the latest research, chitin-bentonite hybrid confirmed the higher adsorption capacity than the pure chitin and bentonite as an adsorbent [15]. Based on the better properties as an adsorbent, this study investigated the use of CB hybrid as a supporting matrix in the immobilization of *Aspergillus fumigatus α*-amylase by carrier binding through physical adsorption to optimize the enzyme stability improvement.

## 2. Materials and Methods

Local fungal isolate *A. fumigatus* was obtained from the Laboratory of Microbiology, Department of Biology, Lampung University. Meanwhile, bentonite and chitin were purchased from Sigma-Aldrich™, and all chemicals and reagents were of analytical grade.

### 2.1. Research Procedure

The phases of production, isolation, partial purification, and characterization of the soluble and immobilized enzymes were based on a previous study [16]. The crude enzyme was partially purified by fractionation using ammonium sulphate and dialysis [17]. The crude enzyme was partly refined by ammonium sulphate in an ice bath. The crude enzyme was brought to 0–20% saturation with ammonium sulphate. The precipitate was removed by centrifugation at 5,000 rpm for 15–20 min, and then, ammonium sulphate was added to the supernatant to 20–85% saturation [16, 17]. The precipitated protein was collected by centrifugation at 5000 rpm for 15 min at 4°C and dissolved in a minimum volume of phosphate buffer (0.025 M; pH 6.5). Then, the enzyme suspension was dialyzed in a dialysis bag (8000 Da) for 24 h at 4°C against phosphate buffer (0.01 M; pH 6.5) [16, 17]. Meanwhile, the immobilization method was based on Yandri et al. [18].

### 2.2. Assay of *α*-Amylase Activity and Determination of Protein Content

The *α*-amylase activity in partial purification steps was assayed by the Fuwa method using iodine reagent. The Fuwa method was used to determine the *α*-amylase activity based on the amount of substrate reduced for incubation time 10 min. This method was appropriate to assay the *α*-amylase activity in purification steps in which probably other amylases such as *β*-amylase and glucoamylase as an exoacting amylase [5] still exist in the crude enzyme, so that the product was specifically formed by *α*-amylase which breaks down the starch polymer from the middle units in rapid incubation [19]. Otherwise, the *α*-amylase activity in characterization steps was assayed by Mandel's method using dinitrosalicylic acid reagent. Mandel's method was used to determine the *α*-amylase activity based on the amount of reducing sugar (glucose) formed. This method was suitable to assay the purified enzyme which was free from other amylases [20]. In addition, the protein content was determined based on the Lowry method [21].

### 2.3. Chitin-Bentonite (CB) Hybrid Synthesis and Characterization

Chitin (0.5 g) was suspended into 500 mL of 1.0 M acetic acid and followed by stirring for 24 h at 30°C. Then, 5 mL of 1.0% glutaraldehyde was added as a cross-linking agent before adding 5.0 g of bentonite and stirring for 4 h at 60°C to obtain the CB hybrid. The hybrid suspension was neutralized using distilled water, and the CB hybrid was filtered and dried in an oven at 60°C. Furthermore, the dried matrix was ground by a laboratory mill and sieved [22, 23], and CB hybrid powder (0.5 g) was analyzed by FT-IR spectroscopy in the wavenumber range of 4000–650 cm^−1^ to determine the functional groups [15]. Surface morphology of the CB hybrid was characterized at 10.00 kV by scanning electron microscopy (SEM) (ZEISS).

### 2.4. Determination of Binding Buffer pH

The free enzyme (0.5 mL) was immobilized onto 0.2 g CB hybrid using 0.1 M phosphate buffer (0.5 mL) with the following pH variation: 6.5, 7.0, 7.5, and 8.0. Initially, these matrices (0.20 g) were washed 2-3 times using each buffer by centrifugation until pH was reached. Then, 0.5 mL of the free enzyme and 0.5 mL of each buffer were added to the matrices; then, the samples were incubated at 4°C for 30 min and centrifuged for 15 min. About 0 25 mL of the supernatant was taken as the “binding” sample and 0.25 mL as a control, separately. Furthermore, the immobilized enzymes were eluted from each matrix using a 1.0 mL mixture of 0.1 M phosphate buffer pH 5.5 and 1 M NaCl (1 : 1), and the samples were then centrifuged for 15 min. About 0.25 mL of the supernatants was taken as the “elution” samples and assayed by the Fuwa method. Buffer pH with both of the lower enzyme activity in the binding process and the higher activity in the elution process was chosen as the binding buffer pH in the immobilization procedure.

### 2.5. Immobilization of *α*-Amylase onto CB Hybrid

CB hybrid (0.20 g) was stabilized using the binding buffer until the pH was reached, and 0.5 mL of the free enzyme and binding buffer were added to the hybrid matrix. In addition, it was incubated at 4°C for 30 min and centrifuged for 15 min. The supernatant of 0.25 mL was taken as a control, and 0.75 mL of the starch substrate was added to the immobilized enzyme. It was incubated at its optimum temperature for 30 min, and the supernatant was assayed by Mandel's method.

### 2.6. Determination of Optimum Temperature

Determination of optimum temperature was conducted at different incubation temperatures in Mandel's assay as follows: 50, 55, 60, 65, 70, and 75°C for 30 min. The incubation temperature, which gave the highest enzyme activity, was determined as the optimum temperature.

### 2.7. Steady-State Kinetics

Kinetic parameters of Michaelis constant (*K*_*M*_) and the maximum velocity (*V*_max_) were estimated using the Lineweaver–Burk plot from experimental data concerning the effect of the starch substrate against the enzyme activity in the following concentration range: 0.2, 0.4, 0.6, 0.8, and 1.0%. The free and immobilized enzymes were incubated at each optimum temperature for 30 min and assayed by Mandel's method. The enzyme activity is proportional to the rate of an enzyme-catalyzed reaction, and the *K*_M_ value was determined as the optimum substrate concentration [1].

### 2.8. Determination of Thermal Stability

Thermal stability of the enzyme was determined from the retainment of residual activity after inactivation at 60°C during the time (*t*_*i*_) variations of 0, 10, 20, 30, 40, 50, 60, 70, and 80 min [24, 25]. The enzyme activity was assayed by Mandel's method, and these data were used to determine *k*_*i*_, *t*_1/2_, and Δ*G*_*i*_ values.

### 2.9. Determination of *t*_1/2_, *k*_*i*_, and Δ*G*_*i*_

Determination of the thermal inactivation rate constant (*k*_*i*_) and half-life (*t*_1/2_) was calculated using the following first-order enzyme inactivation rate equation:

(1) $$\begin{array}{c} \ln\left(\frac{E_{i}}{E_{o}}\right)=-k_{i}\times t_{i}, \end{array}$$

where *k*_*i*_ is the thermal inactivation rate constant, *E*_*o*_ is the residual activity at *t*_*o*_, *E*_*i*_ is the residual activity at *t*_*i*_, and *t*_*i*_ is the thermal inactivation time [4]. The slope of the graph ln(*E*_*i*_/*E*_*o*_) against *t*_*i*_ is determined as *k*_*i*_.

The free energy due to denaturation (*G*_*i*_) is the energy required for enzyme denaturation from the initial state. The value of Δ*G*_*i*_ was estimated from the following thermodynamic equation:

(2) $$\begin{array}{c} \Delta G_{i}=-RT\;\ln\frac{k_{i}\cdot h}{k_{B}\cdot T}, \end{array}$$

where *ΔG*_*i*_ is the transformation of free energy because of denaturation, *R* is the ideal gas constant, *T* is the thermal inactivation temperature (K), *k*_*i*_ is the thermal inactivation rate constant, *h* is the Planck constant, and *k*_*B*_ is the Boltzmann constant [4].

### 2.10. Reusability Study

The immobilized enzyme reacted with the substrate was washed again using binding buffer by centrifugation, and 0.25 mL of the supernatant was taken as the control. Furthermore, the immobilized enzyme was reacted with a new substrate (0.75 mL) and assayed by Mandel's method.

### 2.11. Statistical Analysis

All measurements were done in duplicate (*n* = 2), and data were reported as mean ± standard deviation (SD). Analysis of variance (ANOVA) accompanied by the student *t*-test (paired two-sample for means) was conducted to identify the significant differences between two replicate samples. The level of significance was set at *p* < 0.05. The null hypothesis had been rejected, and there is no difference between the two replicate measurements.

## 3. Results and Discussion

### 3.1. Characterization of CB Hybrid

**Figure 1:**
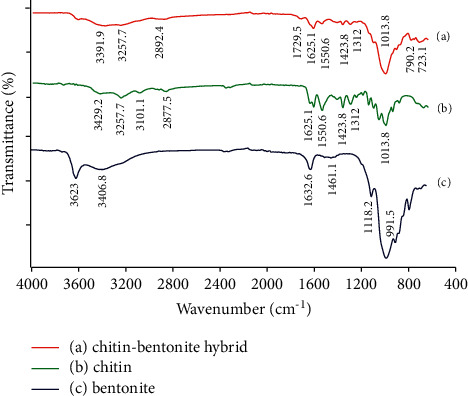
FT-IR spectra of chitin, bentonite, and CB hybrid materials. (a) Chitin-bentonite hybrid. (b) Chitin. (c) Bentonite.

The FT-IR spectra of chitin, bentonite, and CB hybrid are shown in Figure 1. Considering the results of FT-IR, the spectrum of chitin in Figure 1(b) shows the characteristic bands at 3429.2, 3257.7–3101.1, 1625.1, 1550.6, and 1312 cm^−1^. They are attributed to O-H stretching, N-H stretching, and amides I, II, and III, respectively. Another peak at 1013 cm^−1^ corresponded to the C-O stretching in saccharide rings [26]. In contrast, the spectrum of bentonite in Figure 1(c) shows broadband at 3623 cm^−1^ and was assigned to the O-H stretching of the silanol (Si-OH) groups coordinated to octahedral Al^3+^ cations. The HO-H vibration of the adsorbed water molecules was detected at 3406.8 cm^−1^ [27], and the sharp peak in the region of 1118–991 cm^−1^ was associated with the Si-O stretching in the tetrahedral layer [28]. The peak at 723 cm^−1^ confirmed the presence of quartz admixture [29]. On the other hand, the spectrum of CB hybrid is shown in Figure 1(a). As shown in Figure 1(a), the physical interaction between the chitin polymeric chain and bentonite layer cause changes in the corresponding region between 3600 and 3000 cm^−1^. The peak at 3429 cm^−1^ corresponding to the O-H group of chitin was significantly shifted to the lower wavenumber at 3391 cm^−1^. This band shift indicates that both the chitin and bentonite have good physical interaction through intermolecular hydrogen bonds [15]. The bands at 3101 cm^−1^ of the N-H amide groups in a chitin and 3623 cm^−1^ of the O-H group in a bentonite layer disappeared in the spectrum of CB hybrid. These results indicate the existence of good miscibility between chitin and bentonite [30], which confirmed the successful synthesis of the CB hybrid. Meanwhile, the characteristics of each matrix still exist by the existence of some functional groups.

**Figure 2:**
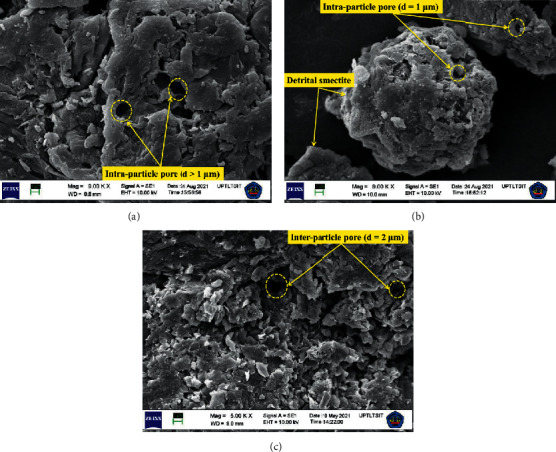
SEM micrographs of (a) chitin, (b) bentonite, and (c) CB hybrid materials.

SEM micrographs of chitin, bentonite, and CB hybrid materials are shown in Figure 2. As a result, surface morphology of CB hybrid in Figure 2(c) appeared with a significant difference compared to bentonite and chitin in Figures 2(a) and 2(b). After modification, the CB hybrid exhibits the larger pores size between the particles compared to bentonite and chitin possibly due to the intercalation of organic moiety. Based on Figure 2(c), at 9000x magnification, the CB hybrid pores size was observed about 2 *μ*m. This leads to increase in the interlayer distance and porous aggregates formation which results in increase of the adsorption of the enzyme [31]. The intercalation of chitin expanded the bentonite basal spacing which facilitated the enzyme adsorption onto CB hybrid. The use of ratio 1 : 1 of chitin and bentonite in this study was suitable to obtain the high intensity of the bentonite basal spacing which indicates the best adsorption capacity of CB hybrid based on the previous research [15].

### 3.2. Determination of Binding Buffer pH

**Figure 3:**
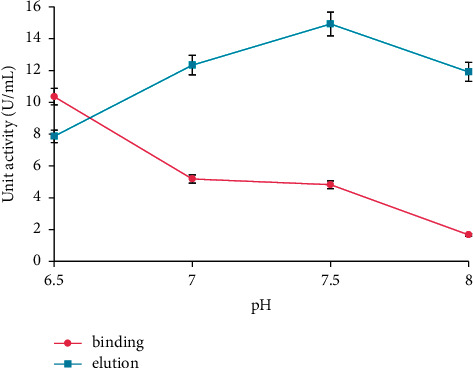
Binding buffer pH for enzyme immobilization. The data are presented as mean ± SD; *n* = 2; *p* ≤ 0.05. The error bars represent standard deviations from two replicates measurements.

Determination of binding buffer pH for enzyme immobilization is shown in Figure 3. The *α*-amylase enzyme molecules can bind onto CB hybrid through physical adsorption in the pH range of 7.0-8.0. pH 7.5 and 8.0 showed the lower enzyme activity in the binding process which indicated that the enzyme had been successfully bound onto the matrix. However, the enzyme activity at pH 8.0 in the elution process was lower than the activity at pH 7.5, in which possibly the enzyme denatured at extreme alkaline pH. In conclusion, pH 7.5 was chosen as the binding buffer pH. In the immobilization process via physical adsorption, the enzyme only can be bound onto supporting matrix at certain pH. The enzyme anionic form exists due to deprotonation of the amine group, while the OH groups in water molecules make the hybrid surface charge positive at higher solution pH [27, 32]. Since oppositely charged groups are due to the electrostatic interaction, the enzyme can be adsorbed to the positively charged active sites of the hybrid surface by the cation-exchange process [33]. At a pH range of 7.0-8.0, enzyme sorption was favored due to the electrostatic attraction between the positive surface charge of hybrid matrix and negative charge on an enzyme molecule. Therefore, the hydrogen bonding between the enzyme molecules and the hybrid matrix can be affected.

### 3.3. Determination of Optimum Temperature

**Figure 4:**
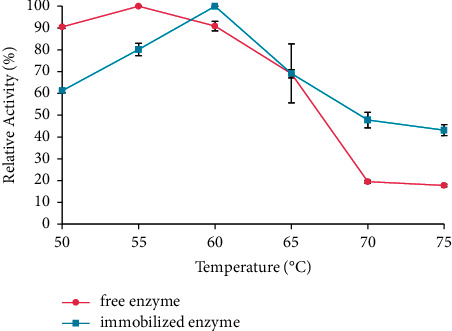
The optimum temperature of the free and immobilized enzymes. The data are presented as mean ± SD; *n* = 2; *p* ≤ 0.05. The error bars represent standard deviations from two replicates measurements.

The temperature profiles of the free and immobilized enzymes are shown in Figure 4. The optimum temperature of the free and immobilized enzyme was 55°C and 60°C, respectively. After immobilization, the enzyme optimum temperature is shifted due to the obstruction of the space by the CB hybrid concerning the enzymatic molecules to prevent denaturation. The kinetic energy will increase with the rate of enzyme-catalyzed reaction at the optimum temperature. The active site of the enzyme will open properly to interact with the substrate after incubation at optimum temperature. Therefore, the activation energy will form the enzyme-substrate complex [34].

### 3.4. Steady-State Kinetics

**Figure 5:**
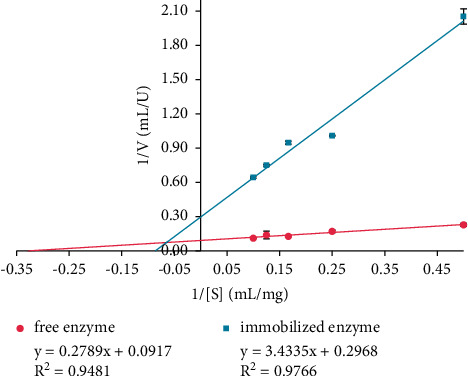
Lineweaver–Burk plot analysis of the enzymatic kinetics of free and immobilized enzyme. The data are presented as mean ± SD; *n* = 2; *p* ≤ 0.05. The error bars represent standard deviations from two replicates measurements.

The Michaelis constant (*K*_*M*_) and maximum velocity (*V*_max_) were determined using the Lineweaver–Burk plot which describes the relationship between substrate concentration and the rate of an enzyme-catalyzed reaction. Meanwhile, the Lineweaver–Burk plot for free and immobilized enzymes is shown in Figure 5.

The Michaelis constant (*K*_*M*_) showed the enzyme affinity for its substrate, while the maximum velocity (*V*_max_) measures the extent. *K*_*M*_ and *V*_max_ values for free and immobilized enzymes are given in Table 1.

The data in Table 1 provide decreasing *V*_max_ and increasing *K*_*M*_ of the immobilized enzyme. The higher *K*_*M*_ of the immobilized enzyme showed a lower affinity. The change in the affinity of the immobilized enzyme due to the conformational change and the lower accessibility to the substrate reduced *V*_max_ of the reaction. The result indicated the higher substrate concentrations required to achieve maximum velocity (*V*_max_) [1, 35]. The immobilized enzyme showed a significant (*p* < 0.05) decrease in its reaction rate compared to the free enzyme. Therefore, the immobilized enzyme showed a significant (*p* < 0.05) increase in thermal stability compared to the free enzyme. Hybrid matrix entrapped the enzyme molecules and prevented the entrance of substrate onto the active site of the enzyme [6]. Furthermore, the hydroxyl groups (O-H) in the hybrid surface strengthened the noncovalent bonds between the enzyme and the matrix [30].

### 3.5. Determination of Thermal Stability

**Figure 6:**
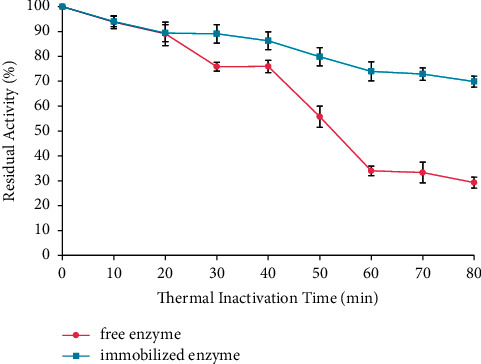
Thermal stability for free and immobilized enzymes. The data are presented as mean ± SD; *n* = 2; *p* ≤ 0.05. The error bars represent standard deviations from two replicates measurements.

The thermal stability for free and immobilized enzymes is shown in Figure 6. The two enzymes were thermal stable after incubation for 80 min; whereas at 60°C, the free and immobilized enzymes lost 72% and 30% of its activity, respectively. Based on the research, the immobilized enzyme molecules were protected by the matrix from the effects of extreme temperature [34]; therefore, it has higher thermal stability than the free enzyme.

### 3.6. Determination of *t*_1/2_, *k*_*i*_, and Δ*G*_*i*_

**Figure 7:**
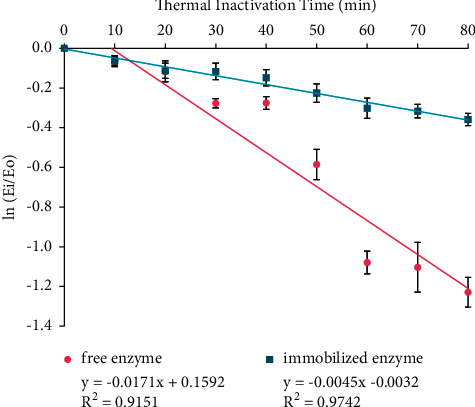
First-order inactivation rate plot for free and immobilized enzymes. The data are presented as mean ± SD; *n* = 2; *p* ≤ 0.05. The error bars represent standard deviations from two replicates measurements.

The residual activities for free and immobilized enzymes from thermal stability assay were plotted to the first-order enzyme inactivation rate graph as shown in Figure 7. The slope of graph ln(*E*_*i*_/*E*_*o*_) against *t*_*i*_ is expressed as a thermal inactivation rate constant (*k*_*i*_). The results showed that *k*_*i*_ values of the free and immobilized enzymes were 0.0171 and 0.0045 min^−1^, respectively. The thermal inactivation rate constant (*k*_*i*_) was used to calculate *t*_1/2_ and Δ*G*_*i*_, as given in Table 2.

The stability improvement of the immobilized enzyme was shown in the decrease of *k*_i_, the increase of its half-life (*t*_1/2_), and the increase of Δ*G*_*i*_ compared to the free enzyme. The decrease in *k*_*i*_ value for immobilized enzyme showed the decrease of denaturation rate due to the flexibility in water. Therefore, the folding conformation in the immobilized enzyme structure will increase to obtain higher stability [14]. The half-life (*t*_1/2_) is the time required for a half inactivation of enzyme activity [4]. Based on the result, the immobilized enzyme takes a longer time to be inactivated and loses its activity; therefore, it is more stable. The immobilized enzyme has higher Δ*G*_*i*_, which indicated the increase of folding conformation in tertiary structure. Furthermore, the increase in Δ*G*_*i*_ caused the enzyme conformation to become more rigid, stable, and less flexible in water. The energy required for enzyme denaturation becomes higher [4]. Based on the increase in half-life, the stability improvement for immobilized *α*-amylase enzyme onto CB hybrid was approximately four-fold higher than the free enzyme.

### 3.7. Reusability Study

**Figure 8:**
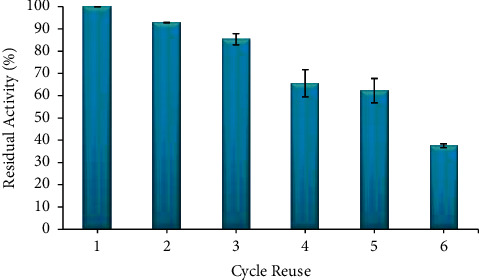
Reuse cycle of the immobilized enzyme. The data are presented as mean ± SD; *n* = 2; *p* ≤ 0.05. The error bars represent standard deviations from two replicates measurements.

Immobilized enzymes can be used repeatedly on new substrates. In the enzyme immobilization through physical adsorption, the molecules cannot interact directly with the substrate [7, 36, 37]. Therefore, the enzyme entrapped onto the matrix can be reacted to the new substrate until all molecules are exhausted. The reuse for immobilized enzyme led to a decrease in enzyme activity, and the cycle is shown in Figure 8.

As a result, the immobilized enzyme onto CB hybrid can be reused in six cycles and still retain 38% of its residual activity. The ratio of reuse and number of cycles for *α*-amylase in other experiments compared to this data is given in Table 3. According to the research, hybrid matrix makes biocatalyst more thermally resistant, stable under reaction conditions, the biocatalytic system becomes reusable and more efficient, improves the purity and quality of products, and protects the enzyme against conformational changes during storage, and the matrix can be more easily separated from the enzyme [7].

## 4. Conclusions

The stability of *A. fumigatus α*-amylase had been improved by the immobilization method onto chitin-bentonite hybrid matrix. The increase of *t*_1/2_ and Δ*G*_*i*_ indicated that the immobilized enzyme become more stable and recyclable biocatalysts. Stability improvement of *α*-amylase enzyme by immobilization onto chitin-bentonite hybrid matrix based on increasing its half-life was approximately four-fold higher than the free enzyme. The residual activity for immobilized enzyme after sixth cycles of reuse was 38%.

## Figures and Tables

**Table 1:** *K*_*M*_ and *V*_max_ values for free and immobilized enzymes.

Sample	*V*_max_ (*μ*mole mL^−1^ min^−1^)	*K*_*M*_ (mg mL^−1^ substrate)
Free enzyme	10.90 ± 1.8940	3.04 ± 1.0430
Immobilized enzyme	3.37 ± 0.1159	11.57 ± 0.7569

The kinetic parameters were determined at each optimum temperature and the starch concentrations from 2.0 to 10.0 mg/mL. The values were shown as mean ± SD, *n* = 2.

**Table 2:** *k*_*i*_, Δ*G*_*i*_, and *t*_1/2_ values of the free and immobilized enzymes.

Sample	*k*_i_ (min^−1^)	*t*_1/2_ (min)	Δ*G*_*i*_ (kJ mole^−1^)	Stability improvement
Free enzyme	0.0171 ± 0.0008	40.53 ± 1.9001	104.47 ± 0.1296	1
Immobilized enzyme	0.0045 ± 0.0004	154.00 ± 13.3963	108.17 ± 0.2389	3.8

The values were shown as mean ± SD, *n* = 2.

**Table 3:** The ratio of reuse and number of cycles for *α*-amylase in other experiments.

Host enzyme	Enzyme type	Matrix type	Reuse cycles	Residual activity (%)	Reference
*A. fumigatus*	*α*-Amylase	Bentonite	6	42	[16]
*B. subtilis* ITBCCB148	*α*-Amylase	Bentonite	5	43	[18]
*B. subtilis*	*α*-Amylase	Chitosan-bentonite hybrid	5	87	[22]
*Saccharomyces cerevisiae*	Yeast alcohol dehydrogenase	Chitin-shellac hybrid	8	46	[38]
Bovine liver	Catalase	Chitosan-bentonite hybrid	20	70	[39]
*Trichoderma harzianum*	*α*-Amylase	PPyAgNp/Fe_3_O_4_ nanocomposite	10	80	[40]
*Thermotoga maritime*	*β*-Glucosidase	Chitin	10	66	[41]
*A. fumigatus*	*α*-Amylase	Chitin-bentonite hybrid	6	38	This study

## Data Availability

The data used to support the findings of this study are available from the corresponding author upon request.
